# Totally Intracorporeal Robot-Assisted Bilateral Ileal Ureter Replacement for the Treatment of Ureteral Strictures using Kangduo Surgical Robot 2000 Plus

**DOI:** 10.1590/S1677-5538.IBJU.2024.0360

**Published:** 2024-08-25

**Authors:** Shubo Fan, Silu Chen, Xinfei Li, Zhihua Li, Kunlin Yang, Han Hao, Liqun Zhou, Xuesong Li

**Affiliations:** 1 Peking University Institute of Urology Peking University First Hospital Beijing China Department of Urology, Peking University First Hospital, Institute of Urology, Peking University, National Urological Cancer Center, Beijing, China

## Abstract

**Purpose:**

Ureteroplasty using buccal or lingual mucosa graft Is feasible for complex proximal ureteral stricture (1, 2). Ileal ureter replacement is considered as the last resort for ureteral reconstruction. Totally intracorporeal robot-assisted ileal ureter replacement can be performed safely and effectively (3). In China, the KangDuo Surgical Robot 2000 Plus (KD-SR-2000 Plus) has been developed featuring two surgeon consoles and five robotic arms. This study aims to share our experience with totally intracorporeal robot-assisted bilateral ileal ureter replacement using KD-SR-2000 Plus.

**Materials and Methods:**

A 59-year-old female patient underwent a complete intracorporeal robot-assisted bilateral ileal ureter replacement for the treatment of ureteral strictures using KD-SR-2000 Plus. The surgical procedure involved dissecting the proximal ends of the bilateral ureteral strictures, harvesting the ileal ureter, restoring intestinal continuity, and performing an anastomosis between the ileum and the ureteral end as well as the bladder. The data were prospectively collected and analyzed.

**Results:**

The surgery was successfully completed with single docking without open conversion. The length of the harvested ileal ureter was 25 cm. The docking time, operation time and console time were 3.4 min., 271 min and 231 min respectively. The estimated blood loss was 50 mL. The postoperative hospitalization was 6 days. No perioperative complications occurred.

**Conclusions:**

It is technically feasible to perform totally intracorporeal robot-assisted bilateral ileal ureter replacement for the treatment of ureteral strictures using KD-SR-2000 Plus. A longer follow-up and a larger sample size are required to evaluate its safety and effectiveness.

**Available at:** http://www.intbrazjurol.com.br/video-section/20240360_Xuesong_et_al

## References

[1] Guliev BG, Komyakov B, Avazkhanov Z, Shevnin M, Talyshinskii A (2023). Laparoscopic ventral onlay ureteroplasty with buccal mucosa graft for complex proximal ureteral stricture. Int Braz J Urol.

[2] Yang K, Fan S, Wang J, Yin L, Li Z, Xiong S (2022). Robotic-assisted Lingual Mucosal Graft Ureteroplasty for the Repair of Complex Ureteral Strictures: Technique Description and the Medium-term Outcome. Eur Urol.

[3] Yang K, Wang X, Xu C, Li Z, Han G, Fan S (2023). Totally Intracorporeal Robot-assisted Unilateral or Bilateral Ileal Ureter Replacement for the Treatment of Ureteral Strictures: Technique and Outcomes from a Single Center. Eur Urol.

